# Determination of Copolymer Composition by Combustion Analysis for Carbon and Hydrogen^1^

**DOI:** 10.6028/jres.064A.015

**Published:** 1960-04-01

**Authors:** Lawrence A. Wood, Irving Madorsky, Rolf A. Paulson

## Abstract

A detailed description is given of the method of combustion analysis for carbon and hydrogen to determine the composition of a copolymer from its carbon-hydrogen ratio. The apparatus and procedures have been developed at the National Bureau of Standards over a period of years. The method has been applied chiefly to determine what fraction of a styrene-butadiene copolymer is derived from styrene. Minor ingredients are removed by extraction, with the exception of the bound mercaptan residue for which correction must be made. The amount of bound mercaptan residue is determined from measurements of the sulfur content of the copolymer by the Carius method. Measurements are made of the oxygen content and the ash content, although these do not enter into the calculations. The standard deviation of a measurement of carbon-hydrogen ratio is approximately 0.0010 and is independent of styrene content. This corresponds to a standard deviation of about 0.036-percent bound styrene for polymers of low styrene content and 0.018 percent for polymers of high styrene content. The accuracy of the method is demonstrated by the fact that observations of carbon-hydrogen ratio for four out of five samples of polybutadiene differed by less than one standard deviation from the theoretical value.

## 1. Introduction

Elemental analysis is probably the most fundamental and unambiguous method of determining the composition of many polymers. When the polymer is a copolymer of two components differing appreciably in carbon-hydrogen ratios, the analyses for carbon and hydrogen by combustion may be used to determine the relative amounts of the two components. It is perhaps the only method that is completely free from possible structural effects. The present work describes in detail the method of combustion analysis used at the National Bureau of Standards to determine the ratio of bound styrene to butadiene in different samples of styrene-butadiene synthetic rubber (SBR, formerly called GR–S). Little modification would be required to apply the method to other hydrocarbon copolymers. Further study of each system would be required to determine its applicability to copolymers containing elements that might cause interference with the determinations of carbon and hydrogen.

References to carbon-hydrogen determinations on polymers in other laboratories are relatively rare. Kemp and Peters [1]^3^ in 1943 at the Bell Telephone Laboratories, in conjunction with their work on unsaturation as determined by iodine chloride addition, published some results of carbon and hydrogen determinations on butadiene-styrene copolymers and used them to make calculations of the bound styrene. No details of their procedure of measurement have been published. With the exception of this publication and two others [2, 3] which give results as a very minor adjunct to other work, we are not familiar with any other carbon and hydrogen determinations on polymers. Consequently, it has seemed desirable to describe in detail the practices employed at the National Bureau of Standards in such determinations.

Carbon and hydrogen determinations have been made with high precision at NBS since about 1929. References to the earlier work are given in a paper by Wagman and Rossini [4]. The method was employed to give information regarding the composition of purified natural rubber by Smith, Saylor, and Wing [5, 6]. The present work was initiated in 1944 as a part of the government synthetic rubber program [7, 8]. Slight modifications and improvements in the method were made over the following years until about 1951. Since then the procedure has remained essentially unchanged.

The procedure is too time consuming and tedious to be applied to routine determinations on samples of only casual interest. It likewise calls for so much care in order to make the determinations with the requisite precision that it can not be recommended for general use. However, it is well adapted to use in measuring the copolymer composition of reference samples intended for standards in developing new procedures. For example, the standards may be used to determine the relationship between the composition and some other property that can be easily measured. In the present instance one of the chief applications of the method was to measure the bound styrene content of samples to be used in determining the relationship of refractive index to the composition of SBR (GR–S) polymers [9 to 11]. Many of these samples were also used in studies of heats of combustion [12], specific heats, and other thermal properties [13 to 15] at NBS. The results of the analyses of these samples have already been reported in the published papers.

## 2. Methods of Calculation

### 2.1. Calculation for a Pure Copolymer

In a copolymer of two hydrocarbon monomers, one of composition C*_j_*H*_k_* and the other of composition C*_p_*H*_q_*, let the percentage of the weight arising from the first monomer be denoted by *X*, and the carbon-hydrogen ratio of the polymer be denoted by *R*. Analysis then requires the calculation of values of *X* from observed values of *R.* By expressing both of these quantities one can derive the following equation:

$$\frac{X}{100}={\left[\frac{jA_{C}}{jA_{C}+kA_{H}}-\frac{pA_{C}}{pA_{C}+qA_{H}}\right]}^{-1}\;\left[\frac{qA_{H}}{pA_{C}+qA_{H}}-\frac{1}{R+1}\right]$$ (1)

where *A*_C_ and *A*_H_, the atomic weights of carbon and hydrogen, are 12.011 and 1.0080, respectively [16].

If the equation is applied to a copolymer of styrene (C_8_H_8_) and butadiene (C_4_H_6_), one has *j* = 8, *k* = 8, *p* = 4, and *q* = 6. The equation then becomes

X=29.0831[11.1809−100/(R+1)]. (2)

For the purpose of calculation of theoretical values, the constants in this equation are given to one more significant figure than would be justified by the probable uncertainty in the accepted atomic weights.

Table 1 shows the values given by this equation for butadiene, styrene, and several of their copolymers. Differentiation of the equation shows that each percent change in styrene content produces a change in carbon-hydrogen ratio ranging from about 0.0275 for polymers of low styrene content to about 0.057 for polymers of high styrene content. In the range of normal SBR, containing about 24-percent styrene, the value is about 0.032. The relation has been presented in graphical form by Kemp and Peters [1].

It is clear that the carbon-hydrogen ratio must be measured with considerable accuracy if an accuracy of a few hundredths of a percent in styrene content is to be obtained. However, there need be no concern for the uncertainty introduced into the calculations by uncertainty in the accepted values of the atomic weights of carbon and hydrogen, since it is of the order of magnitude of hundredths of a percent styrene.

### 2.2. Corrections for Minor Constituents

The discussion thus far has related to an ideal copolymer containing only material arising from the two hydrocarbon monomers. Actual copolymers, especially those made by emulsion polymerization, contain minor constituents. Some of the minor constituents are readily removable by extraction or by solution and precipitation of the polymer. Soap, fatty acid, and stabilizer should normally be removed in this manner so that corrections for their carbon-hydrogen ratios need not be made. The effort spent on such purification is well-justified in terms of increased accuracy. Table 2 shows the carbon-hydrogen ratios of some of these constituents. Special pains should be taken to free the polymer from those minor constituents with carbon-hydrogen ratios differing widely from that of the copolymer. Equal attention should be given to the removal of traces of liquids used in purification.

It should be pointed out that minor constituents containing neither carbon nor hydrogen can be tolerated, since their presence does not affect the carbon-hydrogen ratio. Of course, elements that can cause interference with the actual experimental measurements of carbon and hydrogen should be avoided. Likewise, the addition of oxygen to the polymer at room temperatures may be presumed to be without effect, since there is no evidence of evolution of carbon dioxide or water by the polymer during such oxidation. These facts are the fundamental reason why it is desirable to base calculations of this sort on the carbon-hydrogen ratio rather than the percent carbon or the percent hydrogen in the sample. As would be expected, experimental accuracy is found to be much better when this is done.

According to modern ideas of polymerization [17], the mercaptan used as modifier in the emulsion polymerization of SBR is split into a free radical and a hydrogen ion, one of which initiates and the other terminates a polymer chain. Consequently, the mercaptan is chemically bound in the polymer and cannot be removed by the methods employed to remove the other minor constituents. It has been the practice at NBS to measure the amount of sulfur in the purified polymer and to calculate from it the amount of bound mercaptan. The presence of the sulfur is, of course, of no consequence, as shown by direct experiments mentioned later, but the carbon-hydrogen ratio of the mercaptan assumed to be bound in the polymer is even lower than that of polybutadiene, as may be noted in table 2. The carbon content and the hydrogen content of the portion of the sample consisting of bound mercaptan are subtracted from the directly-observed carbon and hydrogen contents in the calculation of the carbon-hydrogen ratio of the pure copolymer.

The modifier commonly used in butadiene- styrene emulsion polymerizations at 50° C is a mixture of mercaptans, with the major constituent *n*-dodecyl mercaptan. Next in importance in the mixture are tetradecyl and decyl mercaptans. It has been found sufficiently accurate for present purposes to make calculations as if the mixture consisted entirely of dodecyl mercaptan. For low temperature (i.e., 5° C) polymerizations the modifier most commonly used is Sulfole B–8. This is chiefly tertiary dodecyl mercaptan, which of course has the same elementary composition as the *n*-dodecyl mercaptan.

## 3. Apparatus and Procedure

### 3.1. Apparatus

The apparatus used for the combustion was developed from that used in the analysis of benzoic acid by Wagman and Rossini [4]. In the present work the polymer is burned in a stream of oxygen in a quartz combustion tube. That portion of the tube which is in the combustion furnace is packed with cupric oxide wire. The oxygen is first freed of combustible impurities, carbon dioxide, and water by passage through a combustion tube and a train of Ascarite, magnesium perchlorate, and phosphorus pentoxide before it comes into contact with the polymer.

Each sample is placed in a small Pyrex test tube capped with platinum gauze. This arrangement controls the rate of combustion much more satisfactorily than is possible with a platinum boat. This control is especially important when the styrene content of the copolymer is low.

The furnace is a common commercial type with fixed and movable electrical heating units.

The products of combustion are collected in absorption tubes of a design [18] differing from the U-type formerly employed. One of the tubes is packed with magnesium perchlorate and phosphorus pentoxide to absorb the water formed in the combustion. The other tube is packed with Ascarite to absorb carbon dioxide, and with magnesium perchlorate and phosphorus pentoxide to retain water liberated by the reaction of the carbon dioxide with the Ascarite. Weighings are made to hundredths of a milligram on a high-precision analytical balance.

### 3.2. Preparation of Polymer Sample

Purification of the sample of polymer is of the highest importance, as already mentioned. The purification procedure now employed was developed in the course of the present work. The later stages of the development and the application to the samples here described were carried out by Max Tryon. The polymer is first dissolved in benzene and the solution is added dropwise to well-stirred methyl alcohol to coagulate the polymer. The coagulated polymer is then redissolved in benzene. The solution and precipitation processes are carried out three times. This procedure serves to eliminate fatty acids and their salts, stabilizer, and some of the low-molecular-weight polymer. The solution of polymer in benzene is finally frozen by the use of solid carbon dioxide. The refrigerant is then removed and the benzene is sublimed from the system at a low pressure produced by a vacuum pump. This process yields a spongy mass of polymer, which is stored at a low temperature in a vacuum desiccator over magnesium perchlorate until it is cut up for individual analyses. Any possible oxidation during storage, should be at a minimum under these conditions.

### 3.3. Procedure

A sample is removed from the desiccator and cut into small pieces. Eight or more Pyrex test tubes (75-mm long, 12-mm in diam) are cleaned, dried, and weighed. All of the test tubes are filled at the same time with samples weighing between 0.5 and 2 g. The filled test tubes are dried in the vacuum desiccator for 2 hr to remove moisture picked up during the cutting and filling. They are then weighed. Samples are prepared at the same time for determination of sulfur and oxygen.

When a determination is begun the preheater and combustion coils are turned on and the flow of oxygen is adjusted to 250-ml per min. When the fixed unit of the furnace has reached its operating temperature of 800° C, a filled test tube is capped with a piece of platinum gauze, placed on a support of platinum gauze, and is inserted, capped end first, into the combustion tube until the capped end is 6 cm from the end of the fixed heating unit. The combustion tube is swept out with oxygen for 20 min and then the weighed absorption tubes are attached. The absorption tubes are flushed with hydrogen prior to each weighing in order to decrease the magnitude of the correction made for the increase in volume of the solid phase, with resulting decrease in the volume of the gas phase, during the run. Each tube is weighed against a counterpoise of a similar closed absorption tube filled with hydrogen. The movable heating unit is then turned on and pushed forward over the sample until it is 5 cm from the fixed unit. An asbestos pad is inserted between the units.

The temperature of the movable unit is raised rapidly to 300° C and then slowly to 400° C. If the latter temperature is approached too rapidly or if it is exceeded, the polymer sample usually decomposes rapidly, flashes, and burns with violence. Rapid decomposition is indicated by oscillation of the mercury in the flowmeter. If the temperature of 400° C is approached too slowly, carbon forms in the test tube and can be oxidized only at a very slow rate. When conditions are satisfactory, combustion is complete in about 1½ hr. With proper control of temperature some samples are completely decomposed without ignition. Others flash lightly and afterward burn with a small flame at the mouth of the test tube. Under the best conditions no free carbon appears.

After the initial combustion the temperature is slowly increased to 600° C. This is sufficiently high for complete pyrolysis and not high enough to cause softening of the Pyrex test tube. Under normal conditions of slow combustion there is no need to cool the trap that condenses water. Five hours after the absorbers are attached the flow of oxygen is increased to 400 ml per min. Hot water is placed around the water trap and is removed. This operation is repeated several times until the condensed water has disappeared. One hour later the asbestos pad is removed and the movable heating unit is pushed against the fixed unit. The absorbers remain attached for 7 hr. They are then removed and flushed with hydrogen. After grease is dissolved from the spherical glass joints with ether, the absorber and counterpoise are placed in the balance case. They are weighed on the following morning.

The amounts of sulfur in the polymers studied were too low to affect significantly single determinations of carbon and hydrogen, as shown by check experiments utilizing mixtures of benzoic acid with a few tenths of a percent of sulfur. However, when a combusion tube had been used for a long period, it was found that traces of sulfuric acid might collect in the trap that condenses water. In this case, water was retained and the amount of hydrogen found in an analysis was lower than that in the polymer. When the presence of sulfuric acid was suspected, the combustion tube was cleaned and repacked.

### 3.4. Subsidiary Measurements

In the determination of sulfur, a 0.2-g sample of the purified polymer is decomposed by the Carius method [19, 20]. The sulfur is precipitated and weighed as barium sulfate by the conventional microtechnique. The Parr bomb method did not prove to be as reproducible as the Carius method. The results of the sulfur determination are used to calculate the bound mercaptan content of the co-polymer, as discussed in an earlier section.

The amount of oxygen in the polymer is measured by the modified Unterzaucher method developed by Walton, McCulloch, and Smith [21]. The oxygen is converted to carbon monoxide, which is then measured by the use of the NBS colorimetric indicating gel. The ash is determined by simply weighing the sample after the combustion analysis for carbon and hydrogen. The results of the determinations of oxygen and ash are of use in estimating how well the polymer has been purified but do not enter directly into the calculations.

## 4. Results for Compounds of Known Composition

### 4.1. Benzoic Acid

In order to obtain information on the precision and accuracy of the method and to make comparisons with the work of other observers, a series of combustions of purified benzoic acid was made. The benzoic acid used was from a lot of NBS Standard Sample 39f.

Table 3 shows a comparison of values obtained by Wagman and Rossini [4] with those obtained in the first (Madorsky, 1944), and second (Cheek, 1948) stages of development of the present procedure.

It will be noted that the percentage of hydrogen found agrees with the theoretical value within the error of determination but that the percentage of carbon is significantly lower than the theoretical value in all cases. The cause of this discrepancy is not known.

### 4.2. Poly butadiene

In order to obtain further information on the precision and accuracy of the method measurements were made on several samples of polybutadiene. These samples of known composition, of course, possessed a structure nearly the same as the copolymers in which the chief interest was centered. The samples were purified in the manner already described. The results of the analyses are given in table 4. The carbon and hydrogen results are the mean values of five determinations; the sulfur results are the mean of three. Some of these results were utilized in the thermodynamic studies already published [12, 13].

The totals in most cases are about 0.1 percent more than 100 percent. The reason for this is not apparent; it is possible that the mineral contaminants responsible for the ash contain some sulfur or oxygen (perhaps as SO_4_ or CO_3_) which has already been accounted for in the direct analysis for these elements. The oxygen content varies relatively little. The sulfur content depends on the amount of mercaptan used in polymerization and on other polymerization conditions. The ash content, likewise, depends on the conditions of polymerization and purification. It is notably higher for polymer MS–1045, which is the only one shown polymerized at 5° C, where the recipe would be expected to yield a much higher ash content. The sulfur and oxygen are slightly lower than in the others, so that the higher ash does not produce much increase in the total.

Table 5 shows a typical calculation of the correction of measured values of carbon and hydrogen to eliminate the effect of the bound mercaptan. The sample NE–1 polybutadiene—as noted in table 4—was found to contain 0.112 percent sulfur corresponding to 0.707 percent bound mercaptan. This in turn was responsible for 0.503 percent carbon and 0.0915 percent hydrogen. These are subtracted from the observed values, and the carbon-hydrogen ratio of the pure polymer is calculated. The mean value of this ratio *R*′ and those for four other polybutadienes, are given in table 6. The comparison of values shows excellent agreement with the value calculated from the composition—the mean value of all differing by only one in the fifth significant figure. The individual differences from the theoretical value are shown in the last column for comparison with the standard deviations, as calculated from the separate determinations of *R*′ for each sample. In all but one case the error is less than one standard deviation. From statistical theory this situation should occur two-thirds of the time if there are no systematic errors. We conclude that systematic errors are not significant here.

Possible sources of systematic error, if found, would be incomplete extraction of minor constituents (other than bound mercaptan), the presence of residual solvent or precipitant following incomplete drying, and variations in the carbon and hydrogen contents of the bound mercaptan from those calculated for dodecyl mercaptan.

Both the errors and the standard deviations in four out of five cases are smaller than those previously given in table 3 for benzoic acid. This is probably the result of small improvements in technique since 1948. We conclude that the accuracy and precision of the carbon-hydrogen determination are in no way impaired by the use of a well-purified polymer instead of a compound of low molecular weight.

The generally satisfactory nature of the values shown in table 6 for polybutadiene, where the composition is known, gives us confidence in the accuracy of similar values determined for copolymers of butadience with styrene and other monomers, where there are no other satisfactory methods of determining the composition with equal precision.

## 5. Precision of Values for Copolymers

Table 7 shows values of the standard deviation of the carbon-hydrogen ratio *R*′ found for a number of styrene-butadiene copolymers differing in styrene content. The elemental analyses for most of these polymers are given in papers on thermodynamic studies [12, 14, 15]. The values of standard deviation are essentially the same as those shown in table 6 for polybutadiene. No correlation with styrene content or temperature of polymerization is apparent. From the mean value (0.0010) of the standard deviation of *R*′ one may calculate the standard deviation of *X*, the bound styrene content, using the values of the derivative of eq (2) given in an earlier section. One obtains a standard deviation of 0.036 percent bound styrene for polymers of low styrene content or 0.018 percent for polymers of high styrene content. In the range of normal GR–S, containing about 24 percent styrene the value is 0.031.

## 6. Concluding Remarks

The procedure described attains its accuracy and precision by the refinement and improvement of conventional simple operations over a period of years. The trend in analysis recently is, of course, toward the use of rapid physical methods. In a great many instances these involve relative measurements requiring the initial establishment of reference materials with compositions determined by methods such as the one here described, which bases the numbers derived solely on readings of an analytical balance. In fact, as already mentioned, one of the principal applications of the present method has been in the establishment of the relation between refractive index and styrene content for SBR polymers so that refractive index measurements can be used in routine determinations of bound styrene content.

## Figures and Tables

**Table 1:** Theoretical carbon-hydrogen ratios

Butadiene, C_4_H_6_		7. 9438
Butadiene:	Styrene:	
%	%	
80	20	8.5299
79	21	8.5613
78	22	8.5928
77	23	8.6245
76	24	8.6565
75	25	8.6887
Styrene, C_8_H_8_		11.9157

**Table 2:** Theoretical carbon-hydrogen ratios

Dodecyl mercaptan C_12_H_25_SH	5.4995
Phenyl-beta-naphthylamine (C_6_H_5_)NH(C_10_H_7_)	14.6654
Stearic acid CH_3_(CH_2_)_16_COOH	5.9578
Sodium stearate CH_3_(CH_2_)_16_COONa	6.1281
Benzene C_6_H_6_	11.9157
Methanol CH_3_OH	2.9789

**Table 3:** Analysis of benzoic acid

	Wagman and Rossini	Madorsky	Cheek
			
Carbon %	68.8367	68.836	68.8357
Theoretical %	68.8450	68.8450	68.8450
Difference %	−0.0083	−0.009	−0.0093
Standard deviation %	.0013	.0020	.00047
Hydrogen %	4.95461	4.9542	4.9520
Theoretical %	4.95230	4.95230	4.95230
Difference %	+0.00231	+0.0019	−0.0003
Standard deviation %	.0010	.0013	.00045
C/H ratio	13.8931	13.8945	13.9006
Theoretical	13.9016	13.9016	13.9016
Difference	−0.0035	−0.0071	−0.0010
Standard deviation	………………………………	.0048	.0014

**Table 4:** Analysis of polybutadiene

	NE-1	GL-657	36	38	MS-1045
					
	%	%	%	%	%
Carbon	88.656	88.656	88.554	88.486	88.474
Hydrogen	11.1873	11.1795	11.171	11.166	11.1600
Sulfur	0.112	0.072	0.093	0.109	0.083
Oxygen	.145	.145	.16	.145	.12
Ash	.00	.097	.099	.100	.34
					
Total	100.1003	100.1495	100.082	100.006	100.177

**Table 5:** Typical calculation of corrected carbon-hydrogen ratio

(NE–1 polybutadiene)					

Determination	C	C′ = (C–0.503)	H	H′ = (H–0.0915)	R′ = C′/H′
					
	%	%	%	%	
1	88.665	88.162	11.1845	11.0930	7.9475
2	88.663	88.160	11.1919	11.1004	7.9421
3	88.668	88.165	11.1913	11.0998	7.9429
4	88.701	88.198	11.1931	11.1016	7.9446
5	88.583	88.080	11.1758	11.0843	7.9464

Mean					7.9447


**Table 6:** Precision and accuracy of corrected carbon-hydrogen ratios (polybutadienes)

	*R*′	Standard deviation	Difference from theoretical
			
Theoretical	7.9438	………	………
NE–1	7.9447	0.0010	+0.0009
GL–657	7.9432	.0009	−.0006
Sample 36	7.9446	.0014	+.0008
Sample 38	7.9442	.0013	+.0004
MS–1045	7.9426	.0010	−.0012
(5° polymer)			
			
Mean	7.9439	.0011	.0008

**Table 7:** Precision of values for butadiene-styrene copolymers

Designation	Styrene content	Standard deviation of *R*′
		
50° copolymers	%	
GL–658	8.58	0.0009
MS–1726	8.69	.0006
MS–1232	10.71	.0007
MS–1728	11.4	.0016
48–B	55.73	.0006
NE–6	83.67	.0012
5° copolymers		
X–454	8.58	.0010
X–478	22.61	.0011
GL–660	36.26	.0014
GL–661	53.09	.0010
Mean value	…………………………	.0010

## Footnotes

3 Figures in brackets indicate the literature references at the end of this paper.
